# Prognostic Impact of Hyaluronan and Its Regulators in Pancreatic Ductal Adenocarcinoma

**DOI:** 10.1371/journal.pone.0080765

**Published:** 2013-11-11

**Authors:** Xiao-Bo Cheng, Norihiro Sato, Shiro Kohi, Koji Yamaguchi

**Affiliations:** 1 Department of Surgery 1, School of Medicine, University of Occupational and Environmental Health, Kitakyushu, Japan; 2 Department of Breast Surgery, the Fourth Affiliated Hospital of China Medical University, Shenyang, China; The Chinese University of Hong Kong, Hong Kong

## Abstract

**Background:**

Although pancreatic ductal adenocarcinoma is characterized by an abundant stroma enriched with hyaluronan (HA), the prognostic impact of HA and its regulators remains unknown.

**Methods:**

Using immunohistochemistry, expression patterns of HA and its regulators, including a synthesizing enzyme (HAS2), and a degrading enzyme (HYAL1) were investigated in patients who received surgical resection. The prognostic significance of these markers and other clinicopathological variables was determined using univariate and multivariate analyses. The HA levels were determined quantitatively by enzyme-linked immunosorbent assay (ELISA).

**Results:**

We found that strong expressions of HA (P=0.008) and HAS2 (P=0.022) were significantly associated with shorter survival time after surgery. By contrast, weak expression of HYAL1 was significantly associated with poor survival (P=0.001). In multivariate analysis, tumor stage (hazard ratio (HR)=2.76, 95% confidence interval (CI): 1.14-6.66 P=0.024), strong HA expression (HR=6.04, 95%CI: 1.42-25.69 P=0.015), and weak HYAL1 expression (HR=3.16, 95%CI: 1.19-8.40 P=0.021) were independent factors predicting poor survival. ELISA revealed higher concentration of HA in pancreatic cancer tissues than in normal pancreatic tissues (P=0.001).

**Conclusion:**

These findings suggest, for the first time, that HA and its regulators may have prognostic impact in patients with pancreatic cancer.

## Introduction

Pancreatic ductal adenocarcinoma is among the most lethal human malignancies. Despite recent advances in multimodal therapeutic approaches, the fatal prognosis of pancreatic cancer has remained unchanged over the last few decades. Stratification of the patients according to the prognostic information may help with a therapeutic decision and appropriate patient management. Therefore, tremendous efforts have been devoted to identifying novel markers, such as molecular biological markers, to predict clinical outcome and, ultimately, to identify a subset of patients who are more likely to benefit from surgery, chemoradiotherapy, and molecular targeted therapy based on their particular profile [1-4]. 

Hyaluronan or hyaluronic acid (HA) is a well-characterized component of extracellular matrix (ECM), which plays a critical role in a variety of cellular processes. HA regulates cell adhesion, migration, and proliferation by interacting with specific cell surface receptors including CD44 and CD168/RHAMM [5]. HA is synthesized by HA synthases (HAS1, HAS2, and HAS3) [6] and is degraded by hyaluronidases (such as HYAL1) [7]. In normal physiological conditions, the amount of HA is controlled by a balance between synthesis and degradation; however, HA has been shown to be abundantly produced in the surrounding stroma of malignant tumor [8,9]. The HA-rich stroma has been shown to promote tumor progression by enhancing cell proliferation, migration, invasion, metastasis, angiogenesis, and resistance to chemotherapeutic agents [8,9]. 

Previous studies have demonstrated a correlation between HA (and its regulators) and prognosis in a variety of cancers. For example, increased amount of HA in the tumor stroma or in the neoplastic cells themselves predicts poor survival in patients with colorectal cancer [10], gastric cancer [11], breast cancer [12], and prostate cancer [13]. Expressions of HAS1, HAS2 (HA synthases) and HYAL1 (hyaluronidase), have been shown to be associated with diagnosis and prognosis of various tumor types [14-16].

Because pancreatic cancer is characterized typically by a dense desmoplastic stroma, it is highly probable that HA is involved in the malignant properties of this tumor type. Previous studies have shown increased expressions of HA and CD168 in pancreatic cancer [17-20]. More recently, two studies have shown that inhibition of HA by PEGPH20, a HA-targeting enzymatic agent, substantially augments the effect of chemotherapy with gemcitabine in animal models [21,22]. These findings highlight a novel therapeutic approach against pancreatic cancer and suggest that HA and its regulators may play an important role in the aggressive behavior of this highly lethal neoplasm. However, the prognostic relevance of HA and its regulatory components in pancreatic cancer remains unknown. 

In an attempt to determine the prognostic significance of HA in pancreatic cancer, we used immunohistochemical analysis to investigate the expression patterns of HA and its regulators in a series of patients with pancreatic cancer. 

## Patients and Methods

### Ethics statement

A written informed consent was obtained from all patients who approved the use of their tissues for unspecified research purposes and this study was approved by institutional review board of the University of Occupational and Environmental Health. 

### Patients

Surgical specimens were collected from 70 patients with invasive ductal adenocarcinoma of the pancreas who underwent surgical resection at our department between 1982 and 2011. Clinicopathological characteristics of these patients are shown in Table 1. They included 37 men and 33 women with a median age of 68 years (range, 41-89 years). The median observation time for overall survival was 12.2 months (range, 3.1 to 199.9 months). No neoadjuvant radio- or chemotherapy was applied prior to surgical resection in any patient. After resection, 39 patients (56%) were subjected to adjuvant chemotherapy (using gemcitabine, 5-fluorouracil, or tegafur-uracil). All tissues adjacent to the specimens were evaluated histologically according to the criteria of the World Health Organization. The tumor stage was assessed according to the Union for International Cancer Control (UICC). 

**Table 1:** Clinicopathological characteristics of the patients with resected pancreatic cancer (n=70).

Characteristics	Sub-characteristics	No. cases
Age(years)	> 75	16
	≤ 75	54
Gender	Male	37
	Female	33
pT category	pT1	0
	pT2	7
	pT3	42
	pT4	21
pN category	pN0	31
	pN1	39
pM category	pM0	68
	pM1	2
UICC stage	I	5
	II	43
	III	20
	IV	2
Histological grade	Well	16
	Moderate	47
	Poor	4
Lymphatic invasion	Positive	65
	Negative	4
Vessel invasion	Positive	49
	Negative	19
Neural invasion	Positive	60
	Negative	9
Residual tumor category	Positive	30
	Negative	37
Lymph node metastasis	Positive	39
	Negative	31
Chemotherapy	Yes	39
	No	26

### Immunohistochemistry

Five-μm sections of formalin-fixed, paraffin-embedded were sequentially deparaffinized, rehydrated and subjected to antigen retrieval by boiling in a microwave oven for 5 min (pH 6.0,10 mmol/L citrate buffer) three times. After immersing slides in a 3.0% hydrogen peroxidase solution in methanol for 10 min in order to inhibit endogenous peroxidase activity, non-specific binding sites were blocked by pre-incubation with 10% normal goat serum for HA, HYAL1 and 10% normal rabbit serum for HAS2 in 1 mol/L PBS for 10 min at room temperature. The primary antibodies were used at a 1:100 dilution. The slides were incubated at room temperature for 2 hours, with the following primary reagents: anti-HA IgG (polyclonal antibodies, ab53842, abcam, USA); anti-HAS2 IgG (polyclonal antibodies, orb35978, biorbyt, United Kingdom); and anti-HYAL1 IgG (polyclonal antibodies, ab77489, abcam, USA). Sections were incubated for 10 min with rabbit anti-sheep antibody (polyclonal antibodies, ab6747, abcam, USA) for HA, biotinylated anti-goat antibody for HYAL1, biotinylated anti-rabbit antibody for HAS2. Afterwards, the immunoreaction was visualized by using 3,3′-diaminobenzidine staining according to the instructions of the manufacturer. Sections were then counterstained with haematoxylin, dehydrated in graded concentrations of ethanol and mounted. Positive controls for HA, HAS2 and HYAL1 were archival tissues from the chicken cockscomb, normal human brain and normal human liver, respectively.

Stained slides were graded by two individual researchers (N.S., and X.C.) on a blind basis. The staining intensity was determined according to a scoring method described previously by Kramer et al [16] with a minor modification. Briefly, the overall staining intensity was graded (0 to 3+) and was then multiplied by the percentage of positive cells (e.g., 0x100%=0; +1x50%=50; and +3x100%=300). Therefore, each specimen received a staining score between 0 and 300. In an attempt to simply classify the staining pattern, each specimen was divided into two groups: “weak” expression (with a score ranging from 0 to 150) or “strong” expression (with a score ranging from 151 to 300). The number of cases for immunohistochemical evaluation varied from 41 (for HA) to 70 (for HYAL1) because of the different availabilities of archival tissues. 

### Tissue extracts and ELISA assay for hyaluronan

Matched pairs of primary pancreatic tumor and adjacent non-tumor tissues from 11 patients were used for HA concentration assay. Each tissue specimen (0.1g) was homogenized in 500µl Cell Lysis Buffer 2 (R & D Systems, Minneapolis, MN, USA) and 500µl PBS. Samples were incubated overnight at room temperature. The homogenates were clarified by centrifugation at room temperature (1,000×g for 15 min ). The supernatant fraction was aliquoted and stored at -80°C until assayed. HA concentrations in thawed pancreatic tissue extracts were determined by an HA assay kit (R & D Systems, Minneapolis, MN, USA) according to the manufacturer’s instructions. 

### Statistical analysis

All statistical analyses were performed with SPSS statistical software (version 21.0; SPSS, Inc., Chicago, IL, USA). Analyses were performed using only available data; missing information was assumed to be non-informative. Survival curves were constructed with the Kaplan-Meier method and compared by log-rank test. To evaluate independent prognostic factors associated with survival Cox proportional hazards regression analysis was used. Differences in HA levels among pancreatic tissues (normal versus cancer) were compared using the Wilcoxon signed rank test. Statistical differences were considered significant when P-value was less than 0.05. 

## Results

### Immunohistochemistry results

**Figure 1:**
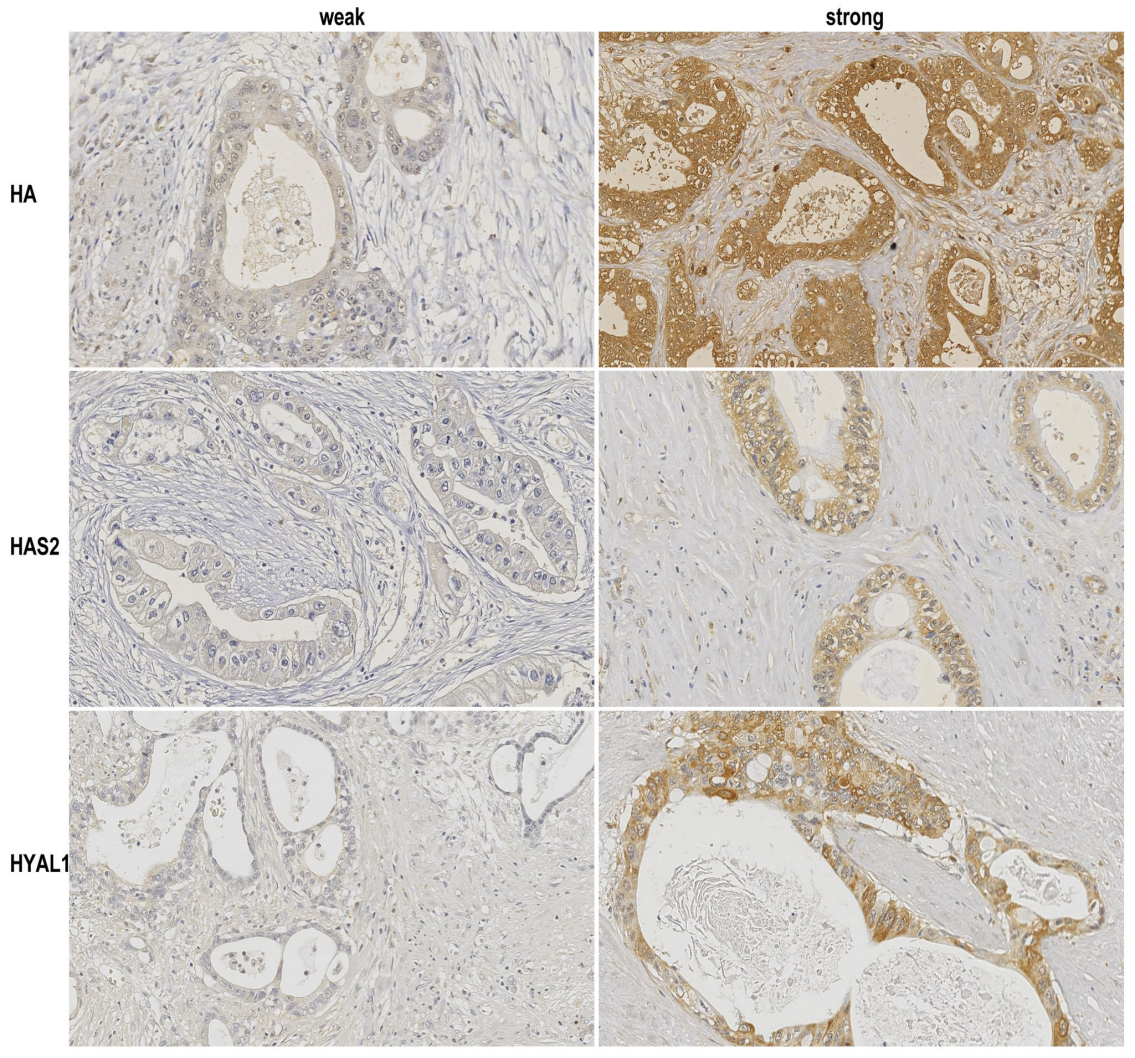
Immunohistochemical stainings of pancreatic cancer tissues with hyaluronan (HA), HAS2, and HYAL1. Stainings of HA, HAS2, and HYAL1 were found in the cytoplasm. (HA) Weak and strong HA staining patterns of tumor and stromal cells in pancreatic cancer. (HAS2) Weak and strong HAS2 staining patterns of tumor cells in pancreatic cancer. (HYAL1) Weak and strong HYAL1 staining patterns of tumor cells in pancreatic cancer. (Original magnification 200×).

HA expression was identified diffusely in both tumor and stromal cells, whereas HAS2 and HYAL1 expressions were observed predominantly in tumor cells. Stainings of HA, HAS2, and HYAL1 were found in the cytoplasm. Of 41 patients evaluable for HA staining, 32 (78%) patients were classified as strong expression for HA, while the remaining 9 (22%) patients were classified as weak, according to the intensity score (Figure 1). Of 66 patients evaluable for HAS2 staining, 52 patients (79%) were classified as strong but the remaining 14 (21%) were classified as weak (Figure 1). Of 70 patients evaluable for HYAL1 staining, 44 patients (63%) were classified as strong and 26 (37%) were classified as weak (Figure 1). The relatively old tissue samples used in this study raise a concern of artifactual degradation of target antigens (proteins) over time. However, the positive rates of these proteins were similar between different time points (1982-1992, 1993-2002, and 2003-2011), suggesting that, despite a long storage duration, antigens for immunostaining are preserved in the archival tissue specimens. 

### Relationship between expressions of HA-family members and survival in patients with pancreatic cancer

**Figure 2:**
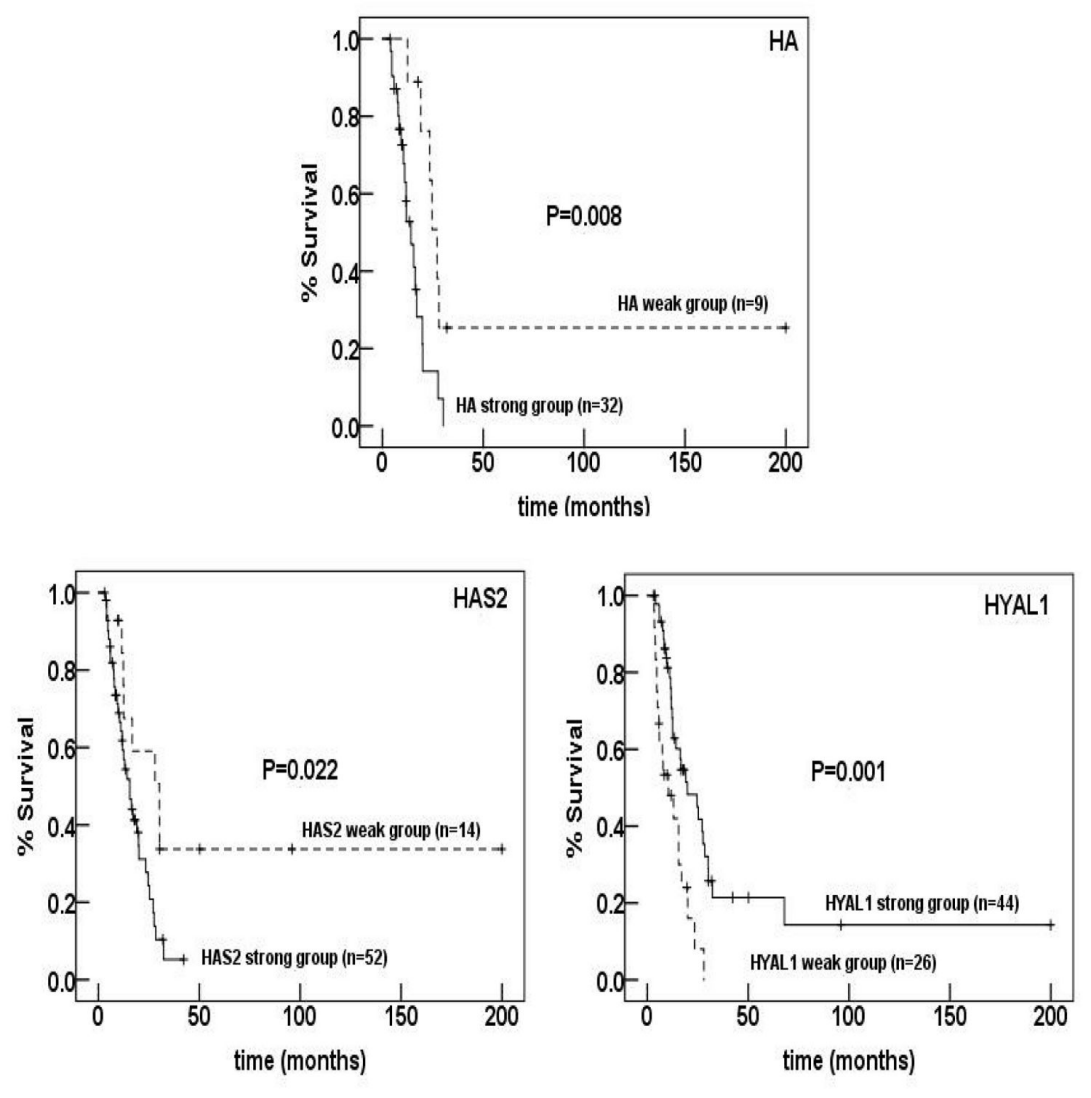
Kaplan-Meier survival curves of patients according to the expression patterns of HA, HAS2, and HYAL1. Strong expressions of HA (P=0.008) and HAS2 (p=0.022) and weak expression of HYAL1 (p=0.001) were significantly associated with poor survival.

We first explored correlations between expression patterns of these proteins and prognosis by comparing survival of patients between strong and weak expression groups. As shown in Figure 2, strong expressions of HA (P=0.008) and HAS2 (P=0.022) were significantly associated with shorter survival time after surgery. By contrast, weak expression of HYAL1 was significantly associated with poor survival (P=0.001).

### Univariate and multivariate analyses for factors affecting survival in patients with pancreatic cancer

To further investigate the prognostic value of these HA-related markers in patients with pancreatic cancer, clinicopathological variables as well as expression status of these markers were analyzed for their correlations with survival using Cox proportional hazard model. As shown in Table 2, univariate analysis revealed significant prognostic factors, including UICC tumor stage, residual tumor, strong HA expression, strong HAS2 expression, and weak HYAL1 expression. Multivariate analysis revealed tumor stage (hazard ratio (HR)=2.76, 95% confidence interval (CI): 1.14-6.66 P = 0.024), strong HA expression (HR=6.04, 95%CI: 1.42-25.69 P=0.015), and weak HYAL1 expression (HR=3.16, 95%CI: 1.19-8.40 P=0.021) (Table 3). 

**Table 2:** Univariate survival analysis of clinical parameters and HA-family members expressions.

Parameters	Hazard ratio	95% confidence interval	P-value
UICC Stage	1.87	1.17 - 2.99	0.009
Largest tumor diameter	1.00	0.97 - 1.04	0.911
Histological grade	0.90	0.51 - 1.56	0.694
Age(> 75 years)	0.576	0.268 - 1.237	0.157
Gender(M)	1.36	0.76 - 2.44	0.302
Chemotherapy(+)	0.700	0.382 - 1.284	0.249
Residual tumor category(+)	2.77	1.43 - 5.37	0.003
Lymph node metastasis(+)	1.26	0.71 - 2.24	0.438
Lymphatic invasion(+)	2.27	0.54 - 9.52	0.261
Vessel invasion(+)	1.13	0.59 - 2.14	0.713
Neural invasion(+)	2.19	0.78 - 6.13	0.135
Strong HA expression	3.49	1.27 - 9.61	0.016
Strong HAS2 expression	2.47	1.12 - 5.45	0.026
Weak HYAL1 expression	2.81	1.51 - 5.22	0.001

**Table 3:** Multivariate survival analysis of conventional prognostic factors and HA-family members expressions.

Factors	Hazard ratio	95% confidence interval	P-value
UICC Stage	2.76	1.14 - 6.66	0.024
Residual tumor category(+)	1.85	0.61 - 5.59	0.277
Strong HA expression	6.04	1.42 - 25.69	0.015
Strong HAS2 expression	3.14	0.31 - 31.72	0.332
Weak HYAL1 expression	3.16	1.19 - 8.40	0.021

### Hyaluronan concentration

**Figure 3:**
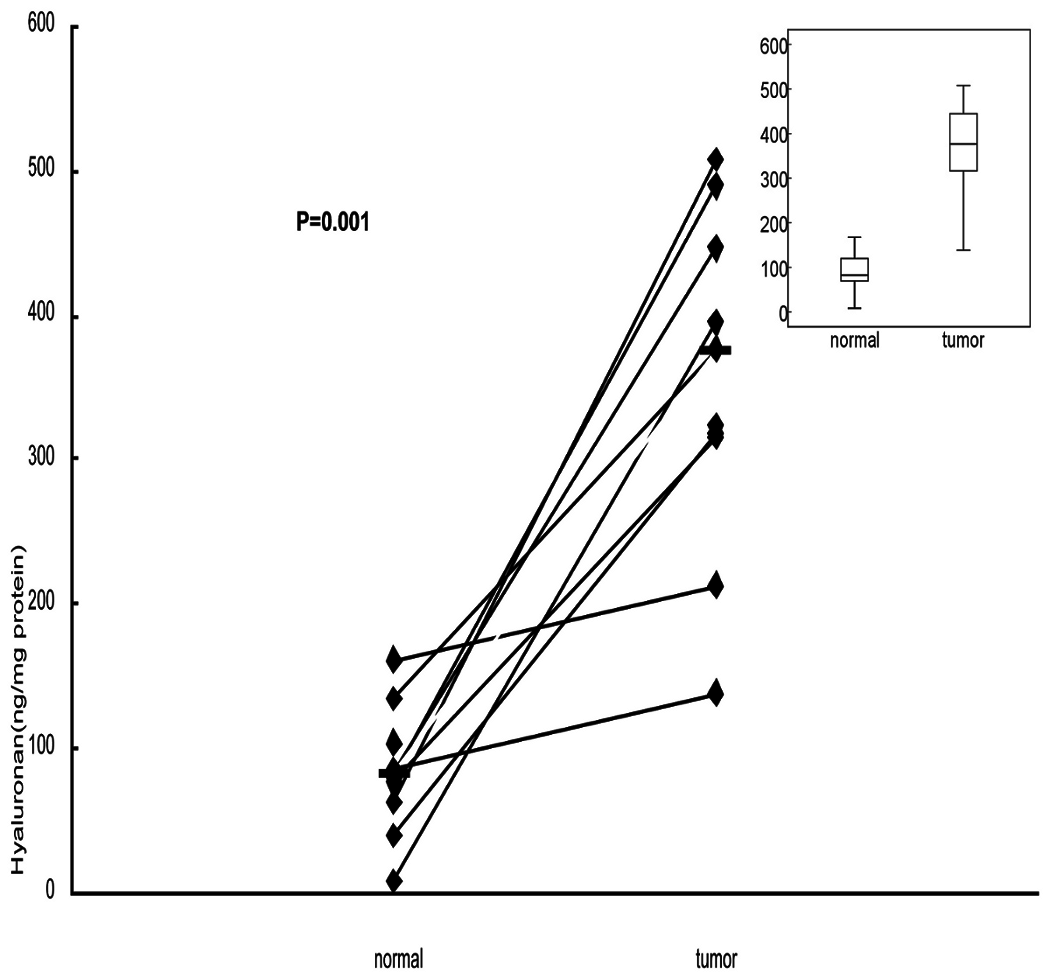
Determination of HA levels in pancreatic tissue extracts. Matched pairs of primary pancreatic tumor and adjacent non-tumor tissues from 11 patients were used for HA concentration assay. The median hyaluronan concentration in normal and tumor tissues were 82.3 ng/mg and 376.7ng/mg, respectively. The concentration of HA in pancreatic cancer tissues is higher than in normal pancreatic tissues (P=0.001; Wilcoxon signed rank test).

We used the HA ELISA assay to measure HA levels (ng/mg) in the extracts from normal pancreatic tissues and pancreatic cancer tissues. As shown in (Figure 3), the HA levels are elevated in pancreatic tumor tissues, regardless of the tumor grade, as compared to those in normal pancreatic tissues. The median hyaluronan concentration in normal and tumor tissues were 82.3 ng/mg and 376.7ng/mg, respectively (P=0.001; Wilcoxon signed rank test).

## Discussion

Increasing evidence has suggested that HA, a major ECM component produced abundantly in tumor stroma, provides a favorable microenvironment for progression of a wide spectrum of human tumors [8,9]. This is particularly true in pancreatic cancer which is characterized by a dense stroma associated with a marked desmoplastic reaction [23]. In the present study, we investigated the prognostic relevance of HA and its major regulators in pancreatic cancer. We demonstrate, for the first time, that expression patterns of HA and its regulators (HAS2 and HYAL1) are significantly associated with survival of patients with pancreatic cancer. Our findings suggest that HA and its regulators can be used in the clinical setting as novel prognostic markers in patients with pancreatic cancer. 

Although UICC stage and residual tumor were also identified as significant prognostic factors in univariate analysis, the P-values for strong HA expression (P=0.015) and weak HYAL1 expression (P=0.021) were lower than those of UICC stage (P=0.024) and residual tumor (P=0.277) in multivariate analysis. These results suggest that, as compared to the UICC stage and residual tumor status, expressions of HA and HYAL1 can be used as more sensitive prognostic markers in pancreatic cancer.

The mechanism by which HA accumulates in cancer stroma has not been yet fully understood, but activation of HA synthesis could contribute to this process. In fact, previous studies have documented increased expressions of HA synthases (HAS1-3) in various cancer types [16,24]. In the present study, we demonstrated a relationship between increased expression of HAS2 and poor prognosis in pancreatic cancer. Interestingly, a previous study used gene expression profiling to demonstrate that HAS2 expression is up-regulated in stromal fibroblasts in response to co-culture with pancreatic cancer cells [25]. This finding suggests that HA can be produced by stromal cells as well as by cancer cells through their crosstalk. 

Unlike HA and HAS2, decreased expression of HYAL1 correlated with poor survival in our present series of pancreatic cancer. HYAL1 is a major hyaluronidase which degrades HA into small fragments [26]. The role of HYAL1 in cancer progression is unclear, but it could be hypothesized that inactivation of HYAL1 can prevent degradation of HA, leading to accumulation of HA in cancer stroma. Interestingly, the gene encoding HYAL1 maps to chromosome 3p21.2-p21.3, a conserved candidate tumor suppressor locus [27], is inactivated in head and neck squamous cell carcinomas [28]. On the other hand, increased expression of HYAL1 has been reported in prostate cancer [29] and breast cancer [30]. These findings suggest that the role of HYAL1 in cancer is tumor-type specific. Our present results raise a possibility of tumor-suppressor role of HYAL1 in pancreatic cancer, but further studies are required to validate its functional relevance. 

Frequent overexpression and accumulation of HA in pancreatic cancer have led to an idea that HA and its regulators could be a therapeutic target. Importantly, HA has been recognized as an attractive target to combat drug resistance, because HA-rich stroma may server as a barrier against the delivery of anticancer drugs to tumor cells [31]. In fact, two recent studies have demonstrated that inhibition of HA by a HA-targeting enzymatic agent robustly enhanced the effect of chemotherapy with gemcitabine in animal models of pancreatic cancer [21,22]. Thus, these findings suggest that HA may serve not only as a prognostic marker but also as an attractive target for pancreatic cancer therapy. 

## Conclusion

The present study demonstrated significant correlations between increased expressions of HA and HAS2, and decreased expression of HYAL1 and poor prognosis in patients with resected pancreatic cancer. These findings suggest an important role of elevated HA production in the aggressive phenotype of pancreatic cancer. 

## References

[1] Jimeno A, Hidalgo M (2006) Molecular biomarkers: their increasing role in the diagnosis, characterization, and therapy guidance in pancreatic cancer. Mol Cancer Ther 5(4): 787-796. doi:10.1158/1535-7163.MCT-06-0005. PubMed: 16648548. 16648548

[2] Ansari D, Rosendahl A, Elebro J, Andersson R (2011) Systematic review of immunohistochemical biomarkers to identify prognostic subgroups of patients with pancreatic cancer. Br J Surg 98(8): 1041-1055. doi:10.1002/bjs.7574. PubMed: 21644238. 21644238

[3] Jamieson NB, Carter CR, McKay CJ, Oien KA (2011) Tissue biomarkers for prognosis in pancreatic ductal adenocarcinoma: a systematic review and meta-analysis. Clin Cancer Res 17(10): 3316-3331. doi:10.1158/1078-0432.CCR-10-3284. PubMed: 21444679. 21444679

[4] Winter JM, Yeo CJ, Brody JR (2013) Diagnostic, prognostic, and predictive biomarkers in pancreatic cancer. J Surg Oncol 107(1): 15-22. doi:10.1002/jso.23192. PubMed: 22729569. 22729569

[5] Turley EA, Noble PW, Bourguignon LY (2002) Signaling properties of hyaluronan receptors. J Biol Chem 277(7): 4589-4592. doi:10.1074/jbc.R100038200. PubMed: 11717317. 11717317

[6] Itano N, Kimata K (2002) Mammalian hyaluronan synthases. IUBMB Life 54(4): 195-199. doi:10.1080/15216540214929. PubMed: 12512858. 12512858

[7] Stern R (2004) Hyaluronan catabolism: a new metabolic pathway. Eur J Cell Biol 83(7): 317-325. doi:10.1078/0171-9335-00392. PubMed: 15503855. 15503855

[8] Itano N, Zhuo L, Kimata K (2008) Impact of the hyaluronan-rich tumor microenvironment on cancer initiation and progression. Cancer Sci 99(9): 1720-1725. doi:10.1111/j.1349-7006.2008.00885.x. PubMed: 18564137. 18564137 PMC11159524

[9] Sironen RK, Tammi M, Tammi R, Auvinen PK, Anttila M et al. (2011) Hyaluronan in human malignancies. Exp Cell Res 317(4): 383-391. doi:10.1016/j.yexcr.2010.11.017. PubMed: 21134368. 21134368

[10] Ropponen K, Tammi M, Parkkinen J, Eskelinen M, Tammi R et al. (1998) Tumor cell-associated hyaluronan as an unfavorable prognostic factor in colorectal cancer. Cancer Res 58(2): 342-347. PubMed: 9443415. 9443415

[11] Setälä LP, Tammi MI, Tammi RH, Eskelinen MJ, Lipponen PK et al. (1997) Hyaluronan expression in gastric cancer cells is associated with local and nodal spread and reduced survival rate. Br J Cancer 79(7-8): 1133-1138. PubMed: 10098747. 10.1038/sj.bjc.6690180 PMC2362238 10098747

[12] Auvinen P, Tammi R, Parkkinen J, Tammi M, Agren U et al. (2000) Hyaluronan in peritumoral stroma and malignant cells associates with breast cancer spreading and predicts survival. Am J Pathol 156(2): 529-536. doi:10.1016/S0002-9440(10)64757-8. PubMed: 10666382. 10666382 PMC1850058

[13] Lipponen P, Aaltomaa S, Tammi R, Tammi M, Agren U et al. (2001) High stromal hyaluronan level is associated with poor differentiation and metastasis in prostate cancer. Eur J Cancer 37(7): 849-856. doi:10.1016/S0959-8049(00)00448-2. PubMed: 11313172. 11313172

[14] Posey JT, Soloway MS, Ekici S, Sofer M, Civantos F et al. (2003) Evaluation of the prognostic potential of hyaluronic acid and hyaluronidase (HYAL1) for prostate cancer. Cancer Res 63(10): 2638-2644. PubMed: 12750291. 12750291

[15] Kramer MW, Golshani R, Merseburger AS, Knapp J, Garcia A et al. (2010) HYAL-1 hyaluronidase: a potential prognostic indicator for progression to muscle invasion and recurrence in bladder cancer. Eur Urol 57(1): 86-93. doi:10.1016/j.eururo.2009.03.057. PubMed: 19345473. 19345473 PMC2828527

[16] Kramer MW, Escudero DO, Lokeshwar SD, Golshani R, Ekwenna OO et al. (2011) Association of hyaluronic acid family members (HAS1, HAS2, and HYAL-1) with bladder cancer diagnosis and prognosis. Cancer 117(6): 1197-1209. doi:10.1002/cncr.25565. PubMed: 20960509. 20960509 PMC3025265

[17] Mahlbacher V, Sewing A, Elsässer HP, Kern HF (1992) Hyaluronan is a secretory product of human pancreatic adenocarcinoma cells. Eur J Cell Biol 58(1): 28-34. PubMed: 1644063. 1644063

[18] Fries H, Elsässer HP, Mahlbacher V, Neumann K, Kern HF (1994) Localisation of hyaluronate (HA) in primary tumors and nude mouse xenografts of human pancreatic carcinomas using a biotinylated HA-binding protein. Virchows Arch 424(1): 7-12. PubMed: 7526947. 7526947 10.1007/BF00197386

[19] Abetamann V, Kern HF, Elsässer HP (1996) Differential expression of the hyaluronan receptors CD44 and RHAMM in human pancreatic cancer cells. Clin Cancer Res 2(9): 1607-1618. PubMed: 9816340. 9816340

[20] Theocharis AD, Tsara ME, Papageorgacopoulou N, Karavias DD, Theocharis DA (2000) Pancreatic carcinoma is characterized by elevated content of hyaluronan and chondroitin sulfate with altered disaccharide composition. Biochim Biophys Acta 1502(2): 201-206. doi:10.1016/S0925-4439(00)00051-X. PubMed: 11040445. 11040445

[21] Jacobetz MA, Chan DS, Neesse A, Bapiro TE, Cook N et al. (2012) Hyaluronan impairs vascular function and drug delivery in a mouse model of pancreatic cancer. Gut 62(1): 112-120. PubMed: 22466618. 22466618 10.1136/gutjnl-2012-302529 PMC3551211

[22] Provenzano PP, Cuevas C, Chang AE, Goel VK, Von Hoff DD et al. (2012) Enzymatic targeting of the stroma ablates physical barriers to treatment of pancreatic ductal adenocarcinoma. Cancer Cell 21(3): 418-429. doi:10.1016/j.ccr.2012.01.007. PubMed: 22439937. 22439937 PMC3371414

[23] Erkan M, Reiser-Erkan C, Michalski CW, Kleeff J (2010) Tumor microenvironment and progression of pancreatic cancer. Exp Oncol 32(3): 128-131. PubMed: 21403605. 21403605

[24] Nykopp TK, Rilla K, Sironen R, Tammi MI, Tammi RH et al. (2009) Expression of hyaluronan synthases (HAS1-3) and hyaluronidases (HYAL1-2) in serous ovarian carcinomas: inverse correlation between HYAL1 and hyaluronan content. BMC Cancer 9: 143. doi:10.1186/1471-2407-9-143. PubMed: 19435493. 19435493 PMC2689240

[25] Sato N, Maehara N, Goggins M (2004) Gene expression profiling of tumor-stromal interactions between pancreatic cancer cells and stromal fibroblasts. Cancer Res 64(19): 6950-6956. doi:10.1158/0008-5472.CAN-04-0677. PubMed: 15466186. 15466186

[26] Csoka AB, Frost GI, Stern R (2001) The six hyaluronidase-like genes in the human and mouse genomes. Matrix Biol 20(8): 499-508. doi:10.1016/S0945-053X(01)00172-X. PubMed: 11731267. 11731267

[27] Csóka AB, Frost GI, Heng HH, Scherer SW, Mohapatra G et al. (1998) The hyaluronidase gene HYAL1 maps to chromosome 3p21.2-p21.3 in human and 9F1-F2 in mouse, a conserved candidate tumor suppressor locus. Genomics 48(1): 63-70. doi:10.1006/geno.1997.5158. PubMed: 9503017. 9503017

[28] Frost GI, Mohapatra G, Wong TM, Csóka AB, Gray JW et al. (2000) HYAL1LUCA-1, a candidate tumor suppressor gene on chromosome 3p21.3, is inactivated in head and neck squamous cell carcinomas by aberrant splicing of pre-mRNA. Oncogene 19(7): 870-877. doi:10.1038/sj.onc.1203317. PubMed: 10702795. 10702795

[29] Lokeshwar VB, Rubinowicz D, Schroeder GL, Forgacs E, Minna JD et al. (2001) Stromal and epithelial expression of tumor markers hyaluronic acid and HYAL1 hyaluronidase in prostate cancer. J Biol Chem 276(15): 11922-11932. doi:10.1074/jbc.M008432200. PubMed: 11278412. 11278412

[30] Tan JX, Wang XY, Li HY, Su XL, Wang L et al. (2011) HYAL1 overexpression is correlated with the malignant behavior of human breast cancer. Int J Cancer 128(6): 1303-1315. doi:10.1002/ijc.25460. PubMed: 20473947. 20473947

[31] Neesse A, Michl P, Frese KK, Feig C, Cook N et al. (2011) Stromal biology and therapy in pancreatic cancer. Gut 60(6): 861-868. doi:10.1136/gut.2010.226092. PubMed: 20966025. 20966025

